# Herpes Simplex Virus Reorganizes the Cellular DNA Repair and Protein Quality Control Machinery

**DOI:** 10.1371/journal.ppat.1001105

**Published:** 2010-11-24

**Authors:** Sandra K. Weller

**Affiliations:** Department of Molecular, Microbial and Structural Biology, The University of Connecticut Health Center, Farmington, Connecticut, United States of America; University of California San Francisco, United States of America

When a virus infects a cell, it must contend with a hostile environment and host machinery that is intrinsically antiviral. One of the hallmarks of herpes simplex virus (HSV) infection is the dramatic reorganization of the infected cell nucleus leading to the formation of large globular replication compartments in which gene expression, DNA replication, and encapsidation occur ([1], [2] and references therein) (see Figure 1). During infection, cellular factors that are beneficial to the virus are hijacked while other factors and pathways are degraded or inactivated. Two of the cellular homeostatic pathways affected by HSV-1 infection are the protein quality control (PQC) and DNA damage response pathways. These events are orchestrated by several viral proteins including the immediate early proteins ICP4, ICP27, ICP0, and ICP22 that allow the virus to create an environment conducive to infection and counteract the cell's intrinsic capacity to inhibit viral infection [3]–[6]. ICP4 and ICP27 play essential roles in stimulation of robust viral gene expression [3], [4]. The immediate early protein ICP0 activates viral and cellular gene expression and functions as an E3 ubiquitin ligase by degrading several cellular proteins [5], [7]. One target of ICP0 is PML, a major component of nuclear foci called ND10 that play a repressive role in viral gene expression. ICP0 interferes with several intrinsic host defense mechanisms including host interferon responses [5], [7], [8], thereby playing a major role in the establishment of a permissive viral infection. ICP22 is required for efficient growth and expression of late viral genes in some but not all cultured cells [6]. ICP22 also plays a role in the post-translational modification of the cellular RNA polymerase II ([9] and references therein). As described below, these immediate early proteins play important roles in remodeling the infected cell nucleus, hijacking host PQC machinery, and manipulating cellular DNA damage responses.

**Figure 1:**
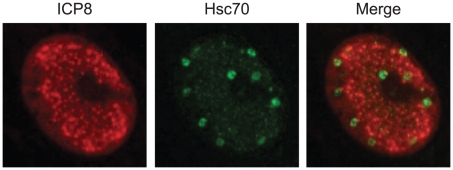
Hsc70 is detected in virus-induced chaperone-enriched (VICE) domains that form adjacent to replication compartments in HSV-1-infected cells. Vero cells were infected with HSV-1 strain KOS at an MOI of 10 for 6 hours. Infected cells were prepared for immunofluorescence imaging of ICP8 in red (left), Hsc70 in green (center), and a merged image (right) as described in [24].

## HSV-1 Infection Results in Remodeling of the Infected Cell Nucleus

The formation of replication compartments follows an ordered assembly process, resulting in a drastic remodeling of the nucleus [1], [2], [10]–[13]. Following entry of viral genomes into the nucleus, nucleoprotein complexes containing viral immediate early proteins ICP4 and ICP27 can be detected on viral genomes [14]. PML and ND10 components are then recruited from ND10 to viral genomes at a position adjacent to the ICP4 foci [14]. The HSV immediate early protein ICP0 counteracts the silencing effects by degrading PML and other ND10 proteins in these newly formed ND10-like foci [15]. Replication proteins including the HSV single-strand DNA binding protein ICP8 are recruited into prereplicative sites that form at a position adjacent to ICP4 nucleoprotein complexes [11], [12]; efficient prereplicative site formation is dependent on the ability of ICP4 to oligomerize on DNA [2]. Prereplicative sites containing replication proteins eventually coalesce into mature replication compartments [16]. In addition to all seven replication proteins, several cellular proteins are also recruited to replication compartments (discussed below). As replication compartments grow and eventually occupy most of the volume of the nucleus, host chromatin is marginalized to the periphery of the nucleus [17], [18]. Other alterations caused by HSV infection include modifications in the nucleolus and disruption of the nuclear lamina [18]–[20].

## HSV-1 Hijacks the Host Protein Quality Control Machinery

Remodeling of the nucleus also involves the reorganization of the cellular PQC machinery, including cellular chaperone proteins (Hsp27, Hsp40, Hsc70, Hsp70, and Hsp90) and components of the 20S proteosome [21], [22]. Within the first 2 hours of infection, Hsc70 and other components of the PQC machinery are recruited into nuclear foci called virus-induced chaperone-enriched (VICE) domains that form adjacent to replication compartments (Figure 1). Viral immediate early proteins have been implicated in the reorganization of PQC machinery into VICE domains; ICP0 and ICP27 are required for robust viral gene expression, and their roles in VICE domain formation may be indirect [23], [24]. On the other hand, ICP22 localizes to VICE domains [25] and appears to be directly required for Hsc70 reorganization [26]. VICE domains form after ND10-like foci are disrupted at a position adjacent to the ICP4 nucleoprotein complexes and prereplicative foci [2]. The precise roles of Hsc70 and VICE domain formation in HSV infection are still a mystery, although the ATPase activity of Hsc70 is essential for efficient immediate early gene expression and replication-compartment formation [23] (C. Livingston and S. K. Weller, unpublished data). We have suggested that Hsc70 may participate in protein remodeling, nuclear transport of viral proteins, and assembly of multi-protein complexes [24]. VICE domains may function as storage sites for chaperones that can rapidly exchange with other sites in the nucleus to achieve protein remodeling. Sequestration of misfolded proteins in VICE domains may also be cytoprotective, and in this light it is of interest to note that they are reminiscent of intranuclear inclusion bodies that form in the presence of excess misfolded protein in some diseases. In cells from patients afflicted with Huntington's disease or spinal cerebellar ataxia, mutant huntingtin and ataxin-1 protein are sequestered, thereby avoiding cytotoxic events that could lead to the induction of apoptosis [27], [28]. Taken together, these results suggest that HSV has evolved to utilize the beneficial aspects of PQC machinery and avoid potentially negative pro-apoptotic consequences related to the presence of misfolded proteins [2], [21], [24].

## HSV Also Manipulates the Cellular DNA Damage Response Machinery

Herpesviruses have also evolved a complex relationship with host DNA damage response pathways (reviewed in [29]–[31]). In response to DNA damage or replication stress, mammalian cells activate signal transduction pathways leading to repair, activation of cell cycle checkpoints, gene silencing or, if the damage is irreparable, induction of apoptosis. DNA damage is generally signaled by the activation of one or more damage-sensing PI3 kinase-like kinases: ATM, ATR, or DNA-PK. Interestingly, in cells infected with HSV-1, some aspects of the damage signaling pathways are activated and others are sabotaged. For instance, DNA-PK is degraded by ICP0, which may inactivate the nonhomologous end-joining pathway at least in some cell types [32]. Interestingly, ATR-mediated phosphorylation of downstream targets RPA and Chk1 is also suppressed [33]–[35]. Furthermore, although some ATM targets are phosphorylated during infection [33], [36]–[38], damage foci that would normally form after DNA damage are prevented due to the ability of ICP0 to degrade cellular histone ubiquitin ligases RNF8 and RNF168 [39]. The observation that ICP0 is responsible for inactivating some aspects of the host damage signaling machinery is consistent with the notion that these pathways may be intrinsically antiviral, resulting in genome silencing, induction of cell cycle checkpoints, or apoptosis. Despite the inactivation of some components of the DNA damage sensing pathways, several of the proteins involved in these pathways are recruited to replication compartments, possibly to aid in viral DNA replication (see below).

## The Formation of Larger-than-Unit-Length Concatemers May Involve Recombination-Dependent Replication Using Viral and Host Proteins

Production of HSV concatemeric DNA is an essential step for the generation of progeny virus, as the packaging machinery must recognize longer-than-unit-length concatemers during encapsidation. Although it has been proposed that the viral genome circularizes and rolling circle replication leads to the formation of concatemers, several lines of evidence suggest that HSV DNA replication is more complex and may involve recombination-dependent replication reminiscent of bacteriophages lambda and T4 (reviewed in [30]). For instance, simple rolling circle replication does not explain the observation that genomic inversions occur as soon as viral DNA synthesis can be detected [40]–[42]. We suggest that HSV has evolved a novel mechanism of DNA replication utilizing viral and cellular proteins.

HSV-1 encodes an enzyme complex reminiscent of the two-subunit Red α/β recombinase encoded by bacteriophage lambda. The viral 5′ to 3′ alkaline exonuclease (UL12) and the single-strand binding protein (ICP8) interact with each other, are recruited to replication compartments, and are essential for efficient virus production ([43] and references within; [N. Balasubramanian and S. K. Weller, unpublished results). UL12 and ICP8 together mediate strand exchange in vitro, suggesting a role as a two-component recombinase reminiscent of the lambda Red α/β recombination system [43]. Alternatively, it is possible that UL12 and ICP8 function to regulate recombination or to process replication intermediates into a form that can be packaged into infectious virus. A role for cellular proteins in viral DNA replication has been suggested by the observation that several cellular factors involved in homologous repair and recombination (HRR) including Mre11, Rad50, Nbs1, and RAD51 are recruited to viral prereplicative sites and replication compartments [33], [36]–[38]. These and other HRR proteins appear to be important for efficient virus production, as mutant or knock down ATM, Mre11, Nbs1, and WRN cell lines exhibit significant defects in virus production [37], [38]. Interestingly, UL12 has recently been shown to interact specifically with the DNA damage sensing MRN complex [44], and Taylor and Knipe have reported that ICP8 interacts either directly or indirectly with 19 cellular proteins involved in DNA replication, repair or recombination [38]. Taken together, these data suggest that viral and cellular proteins collaborate to produce concatemers that can be encapsidated into infectious virions; however, direct proof of this model will require additional experimentation.

## Conclusion: In Order to Create an Environment Conducive to Lytic Infection, HSV-1 Manipulates Several Cellular Homeostatic Pathways

During the earliest stages of viral infection, HSV transforms the cellular environment from one that is hostile to virus infection to one that supports virus growth. Work in this area has only scratched the surface in terms of defining the mechanisms by which ICP0 and other viral proteins manipulate cellular pathways and create environments that promote lytic infection. Elucidating how cellular homeostatic pathways limit viral infections will be important to our understanding of the delicate balance between lytic and latent infection and to aid in the development of new antiviral therapies.
